# Cannabidiol is a partial agonist at dopamine D2High receptors, predicting its antipsychotic clinical dose

**DOI:** 10.1038/tp.2016.195

**Published:** 2016-10-18

**Authors:** P Seeman

**Affiliations:** 1 Department of Psychiatry, University of Toronto, Toronto, ON, Canada; 2 Department of Pharmacology, University of Toronto, Toronto, ON, Canada

## Abstract

Although all current antipsychotics act by interfering with the action of dopamine at dopamine D2 receptors, two recent reports showed that 800 to 1000 mg of cannabidiol per day alleviated the signs and symptoms of schizophrenia, although cannabidiol is not known to act on dopamine receptors. Because these recent clinical findings may indicate an important exception to the general rule that all antipsychotics interfere with dopamine at dopamine D2 receptors, the present study examined whether cannabidiol acted directly on D2 receptors, using tritiated domperidone to label rat brain striatal D2 receptors. It was found that cannabidiol inhibited the binding of radio-domperidone with dissociation constants of 11 nm at dopamine D2High receptors and 2800 nm at dopamine D2Low receptors, in the same biphasic manner as a dopamine partial agonist antipsychotic drug such as aripiprazole. The clinical doses of cannabidiol are sufficient to occupy the functional D2High sites. it is concluded that the dopamine partial agonist action of cannabidiol may account for its clinical antipsychotic effects.

## Introduction

Although currently used antipsychotics for schizophrenia all interfere with the neurotransmission of dopamine,^1^ a report by Leweke *et al.*^2^ showed that cannabidiol at 800 mg/day was as clinically effective as amisulpride in alleviating the signs and symptoms of schizophrenia. In addition, a recent report by P. McGuire *et al.*^3^ found that 1000 mg per day cannabidiol added to the ongoing antipsychotic treatment significantly improved schizophrenia in a study on 88 patients. Because there are no known reports of cannabidiol acting directly on dopamine D2 receptors, the important findings by Leweke *et al.*^2^ and McGuire *et al.*^3^ may indicate that cannabidiol is the first apparent exception to the general rule that all antipsychotics either block or interfere with dopamine at brain dopamine D2 receptors.^4, 5^

In fact, McGuire *et al.*^3^ went so far as to state that the antipsychotic target for cannabidiol was not dopaminergic. Furthermore, the report by Leweke *et al.*^2^ did not attribute the antipsychotic action of cannabidiol to any particular set of receptors in the brain. For example, it is possible that cannabidiol may act on one or more types of receptors to exert its clinical action. Such possible targets include fatty acid amide hydrolase, serotonin-1 receptors, GPR55 receptors and transient potential vanilloid-1 receptors.^6, 7, 8^

Cannabidiol is an active cannabinoid found in high concentration in the cannabis plant (up to 40% in the extract). Cannabidiol has many possible uses in a variety of medical illnesses, especially in certain types of epilepsy and possibly in treating psychosis.^9^

Although dopamine D2 receptors are a main common target for antipsychotic drugs, it was essential, therefore, for this present study to examine whether cannabidiol had any direct action on dopamine D2 receptors that might account for the clinical antipsychotic effects observed by McGuire *et al.*^3^ and Leweke *et al.*^2^ Such an investigation was considered essential in order to test the basis for the commonly known dopamine hypothesis of psychosis,^5^ especially as it is known that the potent cannabinoids HU210 and Win 55,212-2 cause behavioral dopamine supersensitivity and elevated D2High receptors.^5^ That is, should there be no effect of cannabidiol on dopamine D2 receptors, despite having a clinical antipsychotic action, the dopamine hypothesis underlying psychosis would need to be modified.

## Materials and Methods

### Tissue preparation

Rat striatal tissues were used as a source of dopamine D2 receptors. The rat striata were from carbon-dioxide-killed Sprague-Dawley rats or from frozen rat brains (Pel-Freez Biologicals, Rogers, AR, USA). The brain (stored at −70°) was partly thawed and the striata removed. The striata were homogenized in buffer (4 mg frozen tissue per ml buffer), using a teflon-glass homogenizer (with the piston rotating at 500 r.p.m.) and 10 up-and-down strokes of the glass container. The buffer contained 50 mm Tris-HC1 (pH 7.4 at 20 °C), 1 mm EDTA, 5 mm KCl, 1.5 mm CaC1_2_, 4 mm MgC1_2_ and 120 mm NaCl. The homogenate was not washed, centrifuged or preincubated because our previous work found that 30–50% of the D2 receptors were lost by these procedures.^10^

### Cannabidiol/[^3^H]domperidone competition

The dopamine D2 receptors in the rat striatal tissue were measured with [^3^H]domperidone (2 to 3 nm final concentration in the incubation tube; custom-synthesized as [phenyl-^3^H(N)]domperidone; 41.4 Ci mmol^−1^; made by Moravek Biochemicals and Radiochemicals, Brea, CA, USA). Each incubation tube (12 × 75 mm, glass) received, in the following order, 0.5 ml buffer (with or without a final concentration of 10 μm *S*-sulpiride to define nonspecific binding to the dopamine D2 receptors; and with or without 10 μm guanilylimidodiphosphate), 0.25 ml [^3^H]domperidone, and 0.25 ml of tissue homogenate. The tubes (total volume of 1 ml contents) were incubated for 2 h at room temperature (20°), after which the incubates were filtered using a 12-well cell harvester (Titertek, Skatron, Lier, Norway) and buffer-presoaked glass fiber filter mats (Whatman GF/C). After filtering the incubate, the filter mat was rinsed with buffer for 15 s (7.5 ml buffer). The filters were pushed out and placed in scintillation minivials (7 ml, 16 × 54 mm; Valley Container, Bridgeport, CT, USA). The minivials received 4 ml each of scintillant (Research Products International, Mount Prospect, IL, USA), and were monitored 6 h later for tritium in a Beckman LS5000TA scintillation spectrometer (Beckman, Chicago, IL, USA) at 55% efficiency. The specific binding of [^3^H]domperidone was defined as total binding minus that in the presence of 10 μm *S*-sulpiride.

### Analysis and statistics

The competition data were analyzed using a program that provided statistical criteria to judge whether a two-site fit was better than a one-site fit, or whether a three-site fit was better than a two-site fit, using the GraphPad Prism method (GraphPad Software, La Jolla, CA, USA). A total of five completely independent experiments were done. The values for percent inhibition were averaged for each concentration of cannabidiol and the s.e. obtained.

Independently, the Cheng–Prusoff equation (Cheng and Prusoff^11^) was also used to derive the dissociation constants (*K*_i_ values) of the compound from the concentration that inhibited 50% of the high-affinity component (IC_50%_) or 50% of the low-affinity component for [^3^H]domperidone, as indicated in the results. The form of the Cheng–Prusoff equation used was *K*_i_=IC_50_%/(1+C*/*K*_d_), where C* was the final concentration of the radioligand and *K*_d_ was the dissociation constant of [^3^H]domperidone (*K*_d_=0.5 nm), as determined directly by the saturation binding of [^3^H]domperidone (that is, Scatchard plot) to the striatal homogenate. Reagents and compounds were obtained from commercial sources (Sigma-Aldrich, St. Louis, MO, USA).

## Results

The competition between cannabidiol and [^3^H]domperidone at brain dopamine D2 receptors was tested over a range of cannabidiol concentrations from 0.1 to 10,000 nm, using [^3^H]domperidone concentrations (usually 2.3 nm) that occupied about 82% of the D2 receptors. (On the basis of the fact that [^3^H]domperidone had a *K*_d_ of about 0.5 nm for the D2 receptor, the fraction of D2 receptors occupied was *C*/(*C*+*K*_d_) or 2.3 nm/(2.3 nm+0.5 nm)).

**Figure 1:**
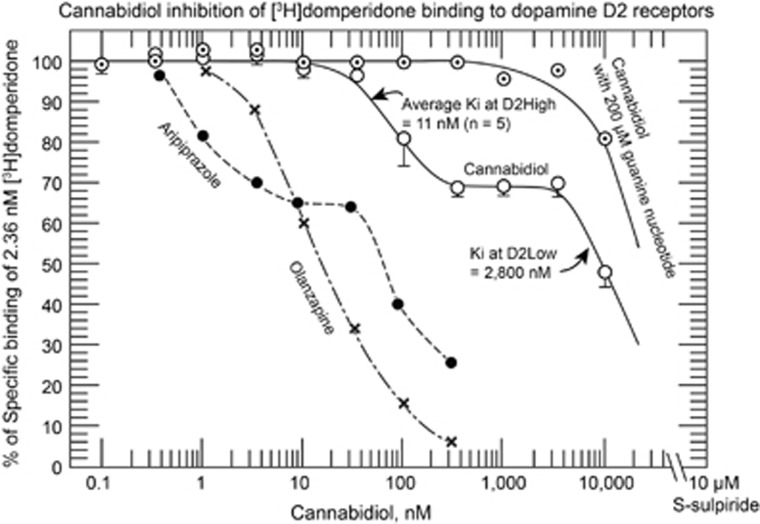
In a biphasic manner, cannabidiol inhibited the binding of [^3^H]domperidone to dopamine D2 receptors in rat brain homogenized striata, similar to the biphasic pattern for other dopamine agonists and the partial dopamine agonist antipsychotic aripiprazole. The earlier published data for aripiprazole^12^ are redrawn here to show the similarity to cannabidiol. Olanzapine (redrawn from Seeman *et al.*^13^) and all other antipsychotic antagonists do not reveal a biphasic pattern when using any antagonist radioligand for D2.

The results for the competition are in Figure 1. The data in Figure 1 show that cannabidiol had a biphasic action in competing against [^3^H]domperidone at the brain dopamine D2 receptors. Cannabidiol inhibited the binding of [^3^H]domperidone at the high-affinity component (at dopamine D2High receptors) with 50% inhibition occurring at 66 nm (*n*=5; s.e. 20 nm). In addition, cannabidiol inhibited the low-affinity component of binding (at dopamine D2Low receptors) with 50% inhibition occurring at 2800 nm (*n*=5).

Using the Cheng–Prusoff relation,^11^ the cannabidiol dissociation constants (*K*_i_ values) were 11 nm at D2High and 2800 nm at D2Low.

Furthermore, in the presence of 200 μm guanilylimidodiphosphate, the binding of [^3^H]domperidone to the high-affinity site (at D2High receptors) was completely abolished. All the dopamine D2High receptors had converted to their D2Low state in the presence of the guanine nucleotide.

In contrast to the reproducible biphasic pattern of cannabidiol competition versus the binding of [^3^H]domperidone, there was no such biphasic competition when cannabidiol competed against the binding of [^3^H]raclopride (data not shown). In fact, cannabidiol inhibited the binding of [^3^H]raclopride with only a single dissociation constant of 4900 nm at the dopamine D2Low receptors (with an IC_50_% concentration of 9000 nm). Such a phenomenon was also previously seen with dopamine, which competed in a biphasic manner versus [^3^H]domperidone but in a monophasic manner at D2Low receptors when using [^3^H]raclopride.^14^

## Discussion

The data in Figure 1 clearly show that cannabidiol inhibited the binding of [^3^H]domperidone in two phases, corresponding to dopamine D2High and D2Low dopamine receptors. This biphasic pattern only occurs with dopamine agonists, as consistently found with hallucinogens^15^ and the commonly used anti-Parkinson agonists.^16^

Such a biphasic pattern does not occur with any of the antipsychotics, except for aripiprazole,^12^ which is a partial agonist (see Figure 1, where the previously published data for aripiprazole^12^ are redrawn to show the similarity to the effect of cannabidiol; olanzapine and all other antipsychotic antagonists do not reveal the biphasic pattern.)^13^ Brexpiprazole and cariprazine are relatively new compounds and have not yet been examined in this assay.

Therefore, the biphasic pattern of cannabidiol in inhibiting the binding of [^3^H]domperidone, in exactly the same way as aripiprazole, indicates that cannabidiol may act clinically as a partial agonist at the dopamine D2 receptors, similar to the clinical antipsychotic action of aripiprazole.

The present data for cannabidiol may help explain some effects of cannabidiol that has actions similar to atypical antipsychotic drugs.^17, 18, 19^

Such a partial agonist-type action at a G-protein-linked receptor has been reported^20^ for delta9-tetrahydrocannabinol at the rat cerebellar cannabinoid receptor but not for cannabidiol. In fact, it was reported^20^ that cannabidiol behaved as an antagonist in the micromolar concentration range.

The present data for cannabidiol acting at the dopamine D2High sites with a dissociation constant of 11 nM may well account for the antipsychotic effects reported by McGuire *et al.*^3^ and Leweke *et al.*,^2^ because the dopamine D2High receptors are considered to be the functional dopamine D2 receptor sites.^21, 22^ More specifically, the clinical daily doses^2, 3^ of 800 mg and 1000 mg cannabidiol would adequately occupy the D2High receptors *in vivo*. For example, a daily dose of 800 mg cannabidiol would result in an extracellular free concentration level in humans of the order of 600 nm, after allowing for at least 99% of the cannabidiol to be bound to plasma proteins.^23^

In addition, considering that cannabidiol has a *K*_i_ value of 11 nm at D2High, as compared with 0.2 nm for aripiprazole, a possible clinical antipsychotic dose for cannabidiol would be of the order of 55-fold higher than aripiprazole. Because the antipsychotic dose of aripiprazole is between 10 and 20 mg per day, the antipsychotic dose for cannabidiol would be 550 to 1100 mg per day, which is the dose range that Leweke *et al.*^2^ and that McGuire^3^ used for patients with schizophrenia.

It may be argued that the present *in vitro* effect of cannabidiol may occur by the action of cannabidiol on the striatal cannabinoid CB1 receptors that are colocalized as heteromers with dopamine D2 receptors,^24, 25^ thereby indirectly influencing the binding of [^3^H]domperidone at the D2 receptors. However, the following important points indicate that such an indirect action through CB1/D2 heteromers is unlikely.

Cannabidiol concentrations between 35 and 350 nm effectively inhibited the binding of [^3^H]domperidone to the D2High site (Figure 1). However, the dissociation constant of cannabidiol at the brain cannabinoid CB1 receptors is between 4350 and 27 542 nm,^9^ indicating that the CB1 receptors would not be significantly affected by cannabidiol concentrations between 35 and 350 nm.

Moreover, although the expression of CB1/D2 heteromers is found in 30 to 60% of monkey striatal neurons,^26^ only a very small non-significant influence was found for the alteration of dopamine competition of the D2 ligand [3H]YM-09151-02 on caudate nucleus membranes by the CB1 agonist CP55940, thereby indicating a very weak interaction between CB1 and D2 receptors.^26^

It is not known why cannabidiol inhibited the binding of [^3^H]domperidone in a biphasic manner at D2High and D2Low receptor sites (Figure 1), yet inhibited the binding in a monophasic manner at only D2Low when using [^3^H]raclopride (data not shown). A similar situation occurred with dopamine,^14^ as mentioned above. It is possible that the two different [^3^H]ligands have a different mode of attachment to the dopamine D2 receptor, based on the fact that domperidone has a pKa of 7.9, whereas raclopride has a pKa of 8.97, resulting in a different proportion of charged and uncharged species of the two ligands, where the charged nitrogen atom of domperidone and raclopride is expected to be the main point of attachment. The fact that [^3^H]raclopride was not competed by cannabidiol at D2High precludes the ready measurement of cannabidiol versus the binding of [^11^C]raclopride in possible studies in humans.

The apparent partial agonist action of cannabidiol *in vitro* in the present work may possibly account for some of the clinical side-effects of cannabidiol such as somnolence, diarrhea, decreased appetite and fatigue.^27^ Moreover, because it is well known that the partial dopamine agonist aripipazole can sometimes elicit psychotic signs and symptoms in patients,^28^ it is entirely possible that long-term use of marijuana can lead to an accumulation of the cannabidiol partial agonist in some individuals and cause a psychotic episode. This is unlikely, however, because cannabidiol has been reported to protect against the effects of tetrahydrocannabinol.^29, 30^
